# *Fusobacterium nucleatum* is associated with worse prognosis in Lauren’s diffuse type gastric cancer patients

**DOI:** 10.1038/s41598-020-73448-8

**Published:** 2020-10-01

**Authors:** Ellen Teresa Boehm, Cosima Thon, Juozas Kupcinskas, Ruta Steponaitiene, Jurgita Skieceviciene, Ali Canbay, Peter Malfertheiner, Alexander Link

**Affiliations:** 1 Department of Gastroenterology, Hepatology and Infectious Diseases, Otto-Von-Guericke University Magdeburg, Leipziger Str. 44, 39120 Magdeburg, Germany; 2 Institute for Digestive Research, Lithuanian University of Health Sciences, Kaunas, Lithuania; 3 Department of Gastroenterology, Lithuanian University of Health Sciences, Kaunas, Lithuania

**Keywords:** Cancer, Gastrointestinal cancer, Gastric cancer, Gastrointestinal cancer

## Abstract

*Fusobacterium nucleatum* (*F. nucleatum*) is frequently detected in primary colorectal cancer (CRC) and matching metastasis, and has been linked to a worse prognosis. We investigated the presence of *F. nucleatum* in gastric cancer (GC) and gastric preneoplastic conditions of the stomach, and its potential prognostic value in GC patients. *Fusobacterium* spp. and *F. nucleatum* were quantified in various specimens from gastrointestinal tract including paired CRC and GC tissues using probe-based qPCR. *Fusobacterium *spp. and *F. nucleatum* were more frequently found in tumorous tissue of CRC and GC compared to non-tumorous tissues. The frequency and bacterial load were higher in CRC compared to GC patients. *F. nucleatum* positivity showed no association to chronic gastritis or preneoplastic conditions such as intestinal metaplasia. *F. nucleatum*-positivity was associated with significantly worse overall survival in patients with Lauren’s diffuse type, but not with intestinal type GC. There was no association with gender, *Helicobacter pylori*-status, tumor stage or tumor localization. However, *F. nucleatum* was positively associated with patient’s age and a trend for a lower global long interspersed element-1 DNA methylation. In conclusion, our work provides novel evidence for clinical relevance of *F. nucleatum* in GC by showing an association between *F. nucleatum* positivity with worse prognosis of patients with Laurens’s diffuse type gastric cancer. Further studies are necessary to explore related mechanistic insights and potential therapeutic benefit of targeted antibiotic treatment in GC patients.

## Introduction

Microbiota of the gastrointestinal tract (GI) is increasingly appreciated in symbiotic relationship with host. GI-microbiota triggers an immune fine-tuning and may play a crucial role in induction of inflammation contributing to a multistep process of carcinogenesis, as proposed for colorectal cancer (CRC) and gastric cancer (GC)^1–4^.

*Fusobacterium nucleatum (F. nucleatum)*, a gram-negative bacterium, is a common member of oral microbiota^5,6^ and has been linked to development of oral plaques and periodontitis^7^. Most intriguingly, it has been suggested to play a role in carcinogenesis as it has been detected in CRC tissues and even cultured from colon biopsies^8^. Recently, *F. nucleatum* has been also detected in several other tumours including oesophageal^9^ and pancreatic cancer tissue^10^.

Most extensive and compelling evidence for the potential role of *F. nucleatum* in carcinogenesis supported by the studies in CRC. *F. nucleatum* is found in tumorous tissues at higher bacterial load in comparison to adjacent non-tumorous mucosa^8,11^. Furthermore, it has been traced from primary tumours to liver metastases and was associated with a worse prognosis, suggesting its potential role not only in carcinogenesis but also possible therapeutic translational implications^12^. For instance, antibiotic therapy of mice with xenograft tumours positive for *F. nucleatum* led to a significant decrease in tumour growth in vivo experiments^12^. From molecular perspective, *F. nucleatum* has been linked to certain molecular alterations in CRC for instance with CpG island methylator phenotype (CIMP), TP53 wild-type, hMLH1 methylation, MSI and CHD7/8 mutation^11,13^. Moreover, *F. nucleatum* has been correlated with expression of proinflammatory genes, lower CD3^+^ T-cell density and increased TNF-α gene expression in CRC^14–16^.

The microbiome composition of the stomach is unique. *Helicobacter pylori* (*H. pylori*) is the predominant species and the key trigger for development of peptic ulcer disease and GC^17,18^. Despite years of research, the exact interaction of *H. pylori* with mucosa remains only partially understood. It is now clearly recognized that *H. pylori* is an infectious disease that causes chronic non-atrophic gastritis (CNAG) that can progress to preneoplastic conditions such as atrophic gastritis (AG), intestinal metaplasia (IM) and finally to dysplasia and cancer^19^. With new sequencing tools, it is increasingly appreciated that not *H. pylori* alone but rather the microbiome in whole complexity contributes to disease conditions. Several studies in detail reported about microbial alterations in stomach^20,21^. *Fusobacterium *spp. are frequently found in stomach mucosa^20–23^. According to few preliminary reports *F. nucleatum* have been found in tumorous GC tissues as well^24,25^, but there are still many unanswered questions. High-throughput techniques including 16 s RNA/DNA sequencing allow only a relative quantification of microbial community while polymerase chain reaction (PCR) based *F. nucleatum* analysis may provide an absolute quantification in relation to human cells. Next, whether *F. nucleatum* may be linked to preneoplastic conditions and contribute to carcinogenesis is still unknown. Most importantly, the clinical and prognostic relevance of *F. nucleatum* in GC has not been studied in detail.

In the present study, we performed in-depth characterization of *Fusobacterium *spp. and *F. nucleatum* in GC. To elaborate on its potential role in gastric carcinogenesis, we evaluated normal gastric mucosa (N), chronic gastritis samples with CNAG or with AG and IM, and correlated the positivity to clinicopathological characteristics and prognosis of GC patients.

## Results

### *F. nucleatum* in CRC

**Figure 1:**
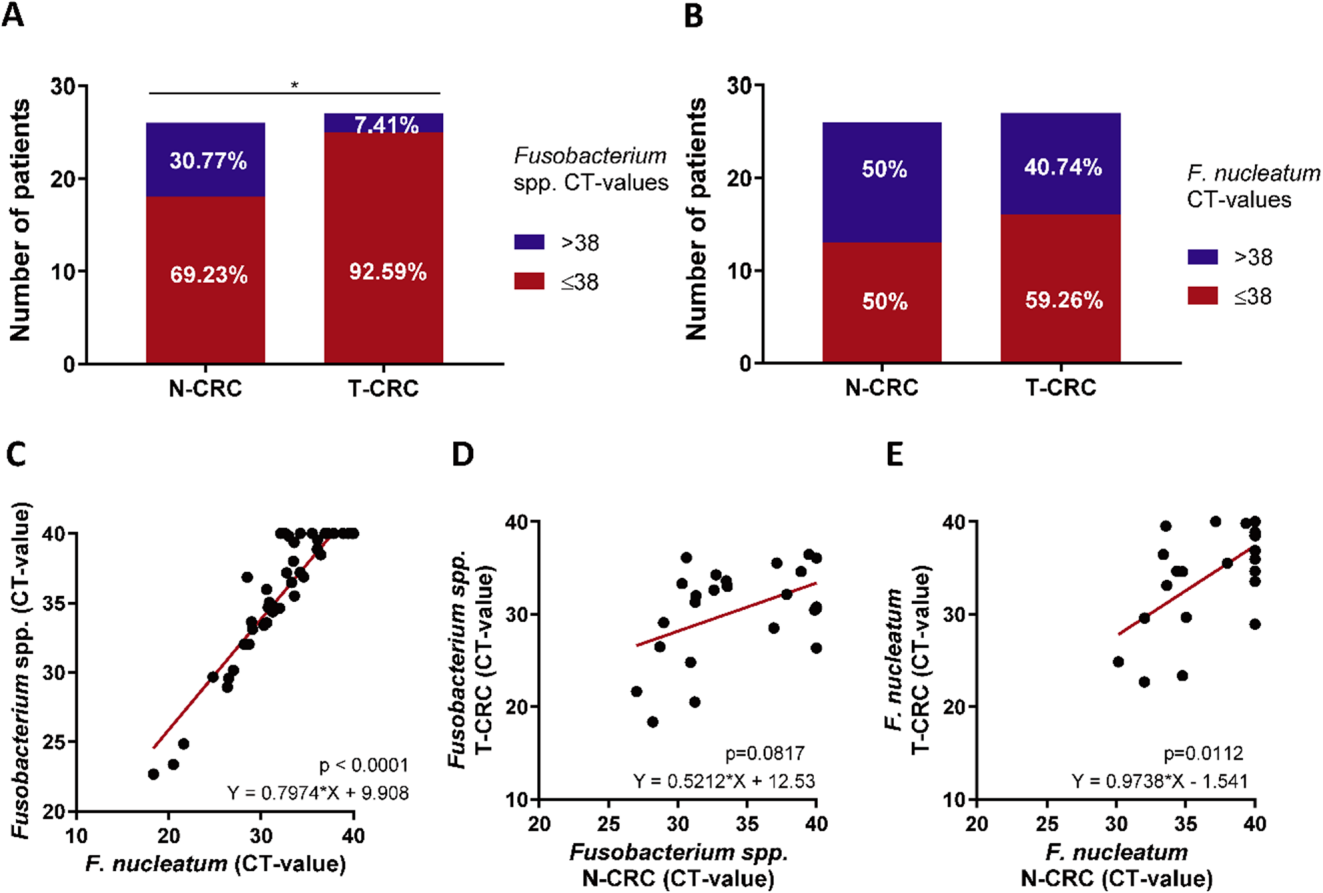
Abundance of *Fusobacterium *spp. and *F. nucleatum* in colorectal cancer patients. (**A**) Proportion of *Fusobacterium *spp. positivity in non-tumorous (N-CRC, n = 26) and tumorous colon tissues (T-CRC, n = 27). (**B**) Proportion of *F. nucleatum* in N-CRC (n = 26) and T-CRC (n = 27) tissues. (**C**) Correlation between *Fusobacterium *spp. and *F. nucleatum* in N- and T-CRC. (**D**) Correlation of *Fusobacterium *spp. abundance between N-CRC and T-CRC. (**E**) Correlation of *F. nucleatum* abundance between N-CRC and T-CRC. Data are presented as raw Ct-values; negative undetectable values were set to Ct of 40, CT-value > 38 were defined as negative and CT-value ≤ 38 were defined as positive. Fisher’s exact and Spearman’s tests were used for analyses.

*F. nucleatum* have been previously evaluated in CRC tissues using PCR-based quantitative analysis. To confirm the analysis in our European cohort, we first validated the quantitative detection method and the reproducibility of *Fusobacterium *spp*. and F. nucleatum* analysis in a subset of samples from CRC patients. Based on our reproducibility results, the cycle threshold (Ct) values of ≤ 38 for both *Fusobacterium *spp. and *F. nucleatum* were classified as positive. In non-tumorous and tumorous CRC tissues we observed *Fusobacterium *spp. positivity in 69.23% (18/26) and 92.59% (25/27) (Fig. 1A, p = 0.0394), respectively. *F. nucleatum* positivity was present in 50% (13/26) N-CRC and 59.26% (16/27) T-CRC specimens (Fig. 1B). Overall, there was a significant correlation between *F. nucleatum* and *Fusobacterium *spp. (Fig. 1C, p < 0.0001). Analysis of the N-CRC and T-CRC samples (Fig. 1D,E) revealed only a trend for positive correlation for *Fusobacterium *spp. abundance in paired samples (p = 0.0817), while *F. nucleatum* load correlated significantly between N-CRC and T-CRC (p = 0.0112). Overall, we confirm that *Fusobacterium *spp. and *F. nucleatum* are more frequently detectable in T-CRC than in N-CRC and *F. nucleatum* load correlates significantly between tumorous and non-tumorous tissues.

### *F. nucleatum* in GC

**Figure 2:**
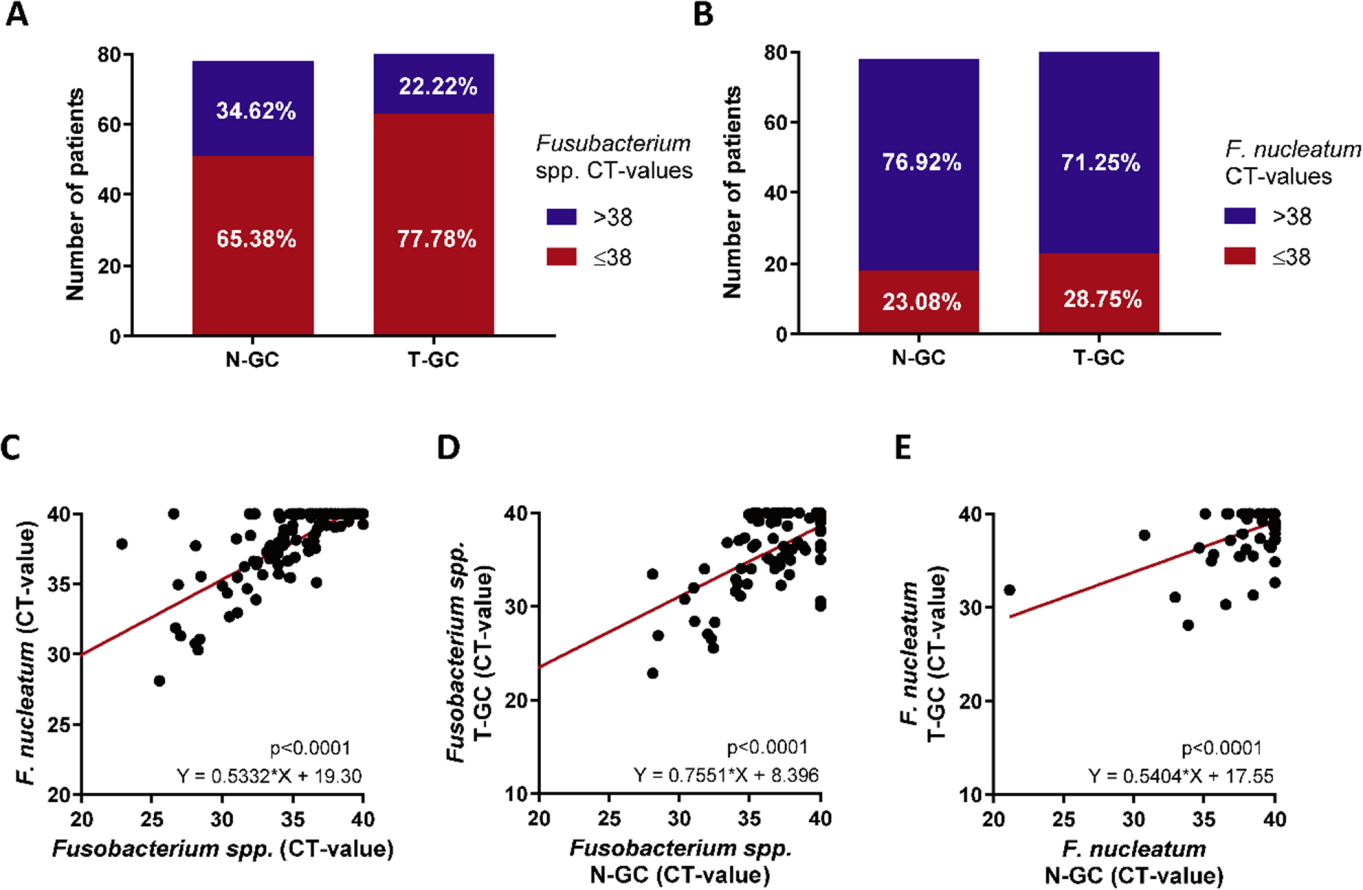
Abundance of *Fusobacterium *spp. and *F. nucleatum* in gastric cancer patients. (**A**) Proportion of *Fusobacterium *spp. positivity in non-tumorous (N-GC, n = 78) and tumorous gastric cancer tissues (T-GC, n = 81). (**B**) Proportion of *F. nucleatum* positivity in N-GC (n = 78) and T-GC (n = 80). (**C**) Correlation between *Fusobacterium *spp. and *F. nucleatum* in N-GC and T-GC. (**D**) Correlation of *Fusobacterium *spp. abundance between N-GC and T-GC. (**E**) Correlation of *F. nucleatum* abundance between N-GC and T-GC. Data are presented as raw Ct-values; negative undetectable values were set to Ct of 40, CT-value > 38 were defined as negative and CT-value ≤ 38 were defined as positive. Fisher’s exact and Spearman’s tests were used for analyses.

Next, we investigated *F. nucleatum* in tumour tissues of gastric cancer (T-GC) and its adjacent mucosa (N-GC). Based on our validation and reproducibility results, the Ct-value of ≤ 38 cycles were defined as positivity also in gastric mucosa. *Fusobacterium *spp. was detectable in 65.38% (51/78) of N-GC and 77.78% (63/81) of T-GC samples (Fig. 2A). *F. nucleatum* was positive in 23.08% (18/78) of N-GC and 28.75% (23/80) of T-GC samples (Fig. 2B). In similar fashion as in CRC, we observed a statistically significant correlation between abundance of *Fusobacterium *spp. and *F. nucleatum* (p < 0.0001) in mucosa of GC patients (Fig. 2C). Furthermore, the abundance of *Fusobacterium *spp. and *F. nucleatum* correlated significantly between N-GC and T-GC (each p < 0.0001, Fig. 2D,E).

### Differences in *F. nucleatum* abundance between CRC and GC

**Figure 3:**
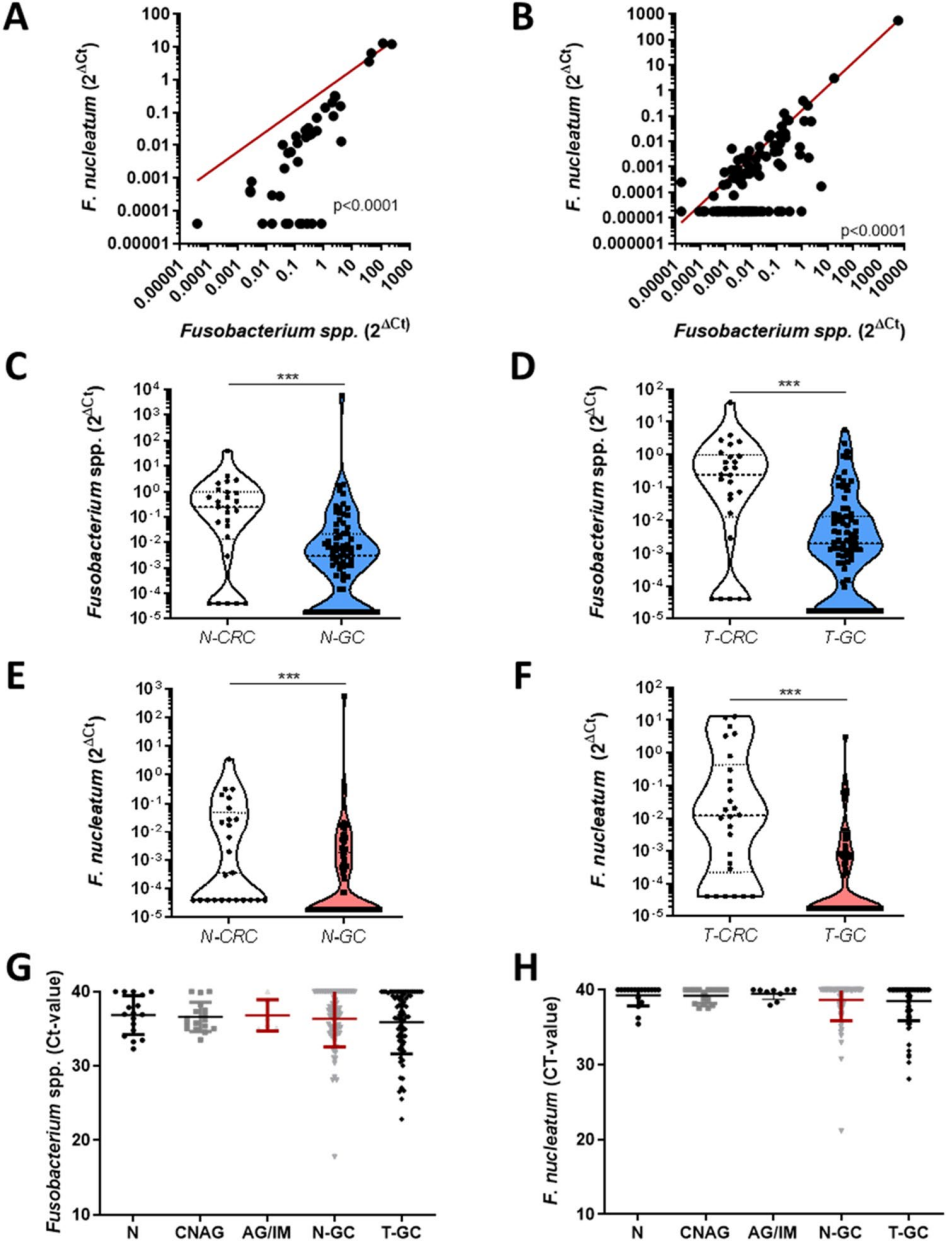
Difference in *Fusobacterium *spp. and *F. nucleatum* between colon, gastric mucosa and abundance in preneoplastic gastric mucosa. (**A**) Correlation between normalized *Fusobacterium *spp. and *F. nucleatum* in N-CRC and T-CRC specimens. (**B**) Correlation between normalized *Fusobacterium *spp. and *F. nucleatum* in N-GC and T-GC specimens. (**C**) Relative abundance of *Fusobacterium *spp. in N-CRC (n = 26) and N-GC (n = 78) (p < 0.0001). (**D**) Relative abundance of *Fusobacterium *spp. in T-CRC (n = 26) and T-GC (n = 79) (p < 0.0001). (**E**) Relative abundance of *F. nucleatum* in N-CRC (n = 25) and N-GC (n = 79) (p < 0.0001). (**F**) Relative abundance of *F. nucleatum* in T-CRC (n = 26) and T-GC (n = 80) (p < 0.0001). (**G**) Abundance of *Fusobacterium *spp. in N (n = 17), CNAG (n = 17), AG/IM (n = 6), N-GC (n = 78) and T-GC (n = 81) tissues in GC (p = 0.97). (**H**) Abundance of *F. nucleatum* in N (n = 18), CNAG (n = 17), AG/IM (n = 9), N-GC (n = 78) and T-GC (n = 80) in GC (p = 0.86). Relative abundance is presented as 2^ΔCT^ values normalized to PGT. Undetectable values were set to the lowest measurable normalized value. Mann–Whitney-test was used for statistical analysis of two groups and Kruskal–Wallis test for more than two groups.

Following normalization to prostaglandin transporter (PGT), we observed a significant correlation between *Fusobacterium *spp. and *F. nucleatum* in CRC (Fig. 3A, p < 0,0001) and in GC (Fig. 3B, p < 0,0001). In comparison to non-normalized values presented in Figs. 1 and 2, the normalized abundance of *Fusobacterium *spp. and *F. nucleatum* was similar between N-CRC and T-CRC and between N-GC and T-GC, respectively (Supplementary Fig. S1). Next, we evaluated the differences in bacterial load of *Fusobacterium *spp. and *F. nucleatum* between CRC and GC. Despite the anatomical distance to oral cavity, abundance of *Fusobacterium *spp. in N-CRC and T-CRC was significantly higher than in N-GC and T-GC, respectively (Fig. 3C,D). In addition, *F. nucleatum* was higher in N-CRC and T-CRC compared to N-GC and T-GC, respectively (Fig. 3E,F).

### *F. nucleatum* in preneoplastic conditions in comparison to GC

To explore the potential involvement of *Fusobacterium *spp. and *F. nucleatum* we compared samples from patients with normal mucosa (N), CNAG, AG/IM, N-GC and T-GC. The analysis of Ct-values, revealed relatively similar pattern of *Fusobacterium *spp. and *F. nucleatum* abundance in normal and chronic gastritis with or without preneoplastic conditions in comparison to GC, suggesting that *F. nucleatum* may be probably involved in rather late stages of classical Correa’s cascade of gastric carcinogenesis (Fig. 3G,H). *F. nucleatum* was present in 16.7% (3/18) of N, 17.65% (3/17) CNAG mucosa and 0% (0/9) in AG/IM mucosa, which was not significantly different to N-GC and T-GC. Since none of the AG/IM mucosa samples were positive for *F. nucleatum,* we did not perform any correlation to OLGA/OLGIM. To evaluate potential association between *F. nucleatum* and *H. pylori* in non-neoplastic mucosa, we compared *Fusobacterium *spp. and *F. nucleatum* levels between subjects with and without active *H. pylori* infection irrespective of gastritis type or severity in total cohort of non-neoplastic mucosa. As shown in the Supplementary Fig. S2, we observed no difference in *Fusobacterium *spp. between *H. pylori* positive and negative gastric mucosa, while a slightly lower level of *F. nucleatum* was found in *H. pylori* positive mucosa (p = 0.046). However, only each three samples from each *H. pylori* positive and negative groups were below the defined Ct-value of 38 and further data are needed to elaborate on this topic.

### *F. nucleatum* and GC subgroup analysis

To evaluate if *F. nucleatum* might be associated with specific GC characteristics, we divided the GC cohort in *F. nucleatum* positive and negative groups (Table 1). We compared the *F. nucleatum-*positive and -negative groups with regard to gender, tumour localization, UICC and TNM stages, grading, Lauren’s classification and *H. pylori* status but except for age there were no differences between the groups.

**Table 1:** Clinicopathological characteristics of Gastric Cancer patients in relation to *F. nucleatum* positivity.

	*F. nucleatum*						
	All		Positive		Negative		*p*
	n = 81	%	n = 23	%	n = 57	%	
Age	65.85	± 11.58	70.04	± 9.70	64.16	± 12.19	0.042*
**Gender**							0.21
Male	47	58	11	48	36	63	
Female	34	42	12	52	21	37	
**Tumor localization**							0.37
Cardia	8	10	4	17	4	7	
Corpus	45	56	12	52	32	56	
Antrum	28	34	7	30	21	37	
**UICC**							0.75
I	16	20	4	17	11	19	
II	21	26	8	35	13	23	
III	36	44	9	39	27	47	
IV	8	10	2	9	6	11	
**T**							0.77
1 + 2	18	22	4	17	13	23	
3	36	45	10	43	26	46	
4	27	33	9	39	18	32	
**N**							0.26
0	29	36	9	39	19	33	
1	15	19	6	26	9	16	
2	13	16	1	4	12	21	
3	23	28	6	26	17	30	
Unknown	1	1	1	4	0	0	
**M**							1
0	72	89	20	87	51	89	
1	8	10	2	9	6	11	
Unknown	1	1	1	4	0	0	
**Grading**							0.37
1	3	4	0	0	3	5	
2	29	36	10	43	18	32	
3	49	60	13	57	36	63	
**Laurén-classification**							0.61
Diffuse Type	44	54	12	52	32	56	
Intestinal Type	26	32	6	26	19	33	
Mixed Type	7	9	3	13	4	7	
Unknown	4	5	2	9	2	4	
***H. pylori***							0.82
Negative	8	10	2	9	6	11	
Positive	17	21	5	22	12	21	
Unknown	56	69	16	70	39	68	

*F. nucleatum* positivity was defined by the cut-off of ≤ 38. *Unpaired t-test. ns: non-significant. *F. nucleatum* data were available only for 80 subjects. UICC: Union for International Cancer Control; T-primary tumor stage; N- lymphnode metastasis staging; M: metastasis staging.

### *Fusobacterium *spp*. and F. nucleatum* correlate with age

**Figure 4:**
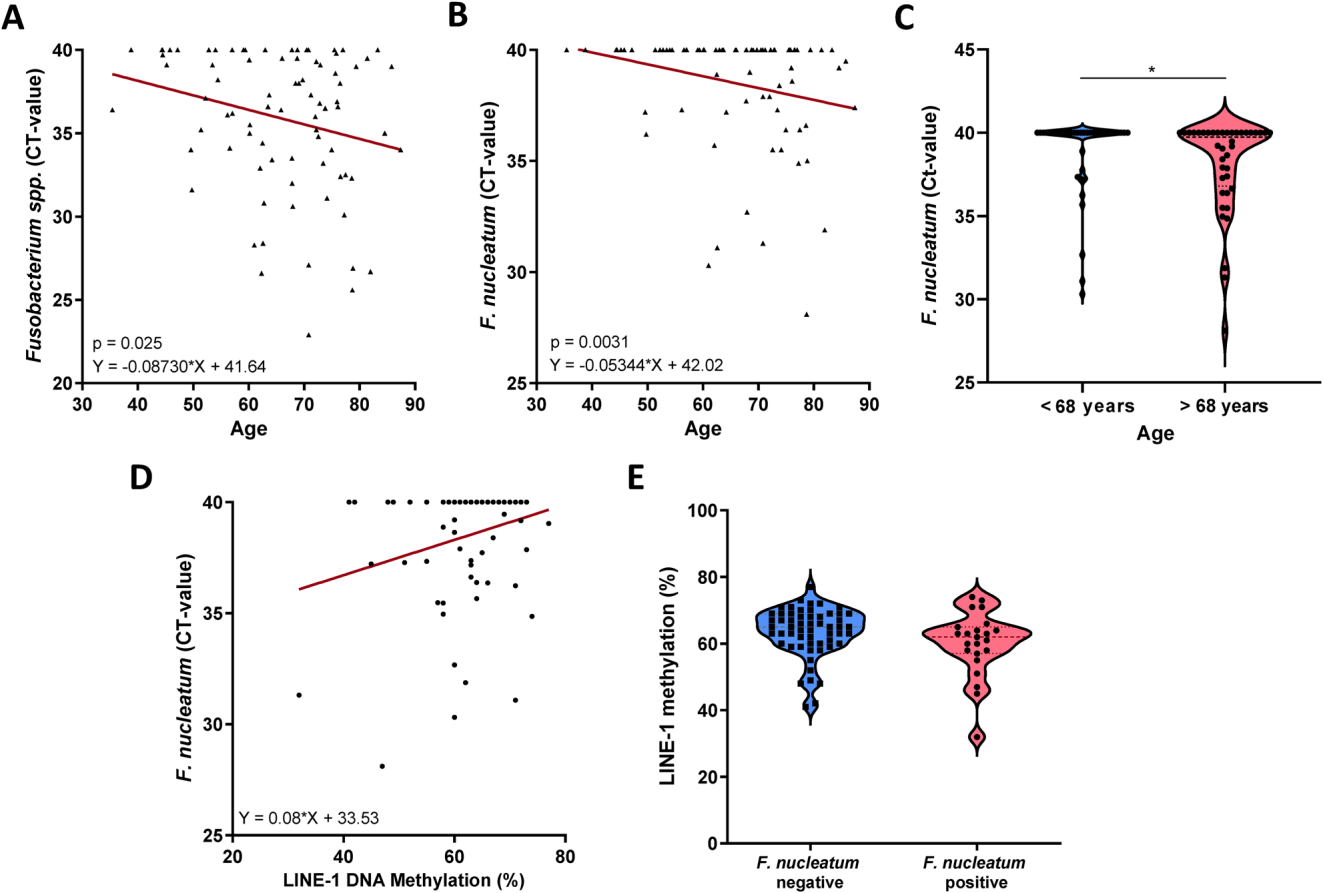
Correlation between *F. nucleatum*, LINE-1- and patients age in tumorous tissue of GC patients. (**A**) Correlation between *Fusobacterium *spp. abundance in T-GC and patients age in GC patients (n = 81, p = 0.025). (**B**) Correlation between *F. nucleatum* abundance in T-GC and patients age in GC patients (n = 80, p = 0.0031). (**C**) Differences in *F. nucleatum* abundance based on the patients age defined as below or above median age (68 years). (**D**) Correlation between *F. nucleatum* and LINE-1 DNA methylation in T-CRC specimens (n = 80, p = 0.153). (**E**) LINE-1 DNA methylation differences between *F. nucleatum-*positive (n = 23) and –negative (n = 57) T-GC (p = 0.09). Mann–Whitney and Spearman’s tests were used for analysis.

Correlation analysis revealed a positive correlation between patient’s age and *Fusobacterium *spp. and between patient’s age and *F. nucleatum.* Older patients had higher *Fusobacterium *spp. (p = 0.025, Fig. 4A) and higher *F. nucleatum* (p = 0.0031, Fig. 4B) abundance in T-GC. Furthermore, patients with *F. nucleatum* positive T-GC were overall older than patients with negative T-GCs. Based on the median age with cut-off of 68 years, older groups with GC had significantly higher *F. nucleatum* load compared to younger patients (Fig. 4C).

### Correlation with global and gene specific methylation changes

It has been recently suggested that *F. nucleatum* may be associated with distinct molecular alterations in cancer. We evaluated possible correlation between *F. nucleatum* and global DNA hypomethylation using surrogate long interspersed element-1 (LINE-1) methylation and miR-137 promoter methylation, which are frequently deregulated in GC and CRC. Overall, correlation analysis between LINE-1 and *F. nucleatum* revealed a non-significant trend for lower LINE-1 methylation in subjects with higher *F. nucleatum* load (p = 0.156, Fig. 4D). LINE-1 methylation in the *F. nucleatum*-positive group was slightly lower as in *F. nucleatum*-negative group although the difference did not reach statistical significance (60.1 ± 9.6 vs. 63.4 ± 7.4, p = 0.09) (Fig. 4E). For comparison, gene specific DNA methylation analysis of miR-137 and *F. nucleatum* revealed no difference between the groups (data not shown).

### Survival analysis

**Figure 5:**
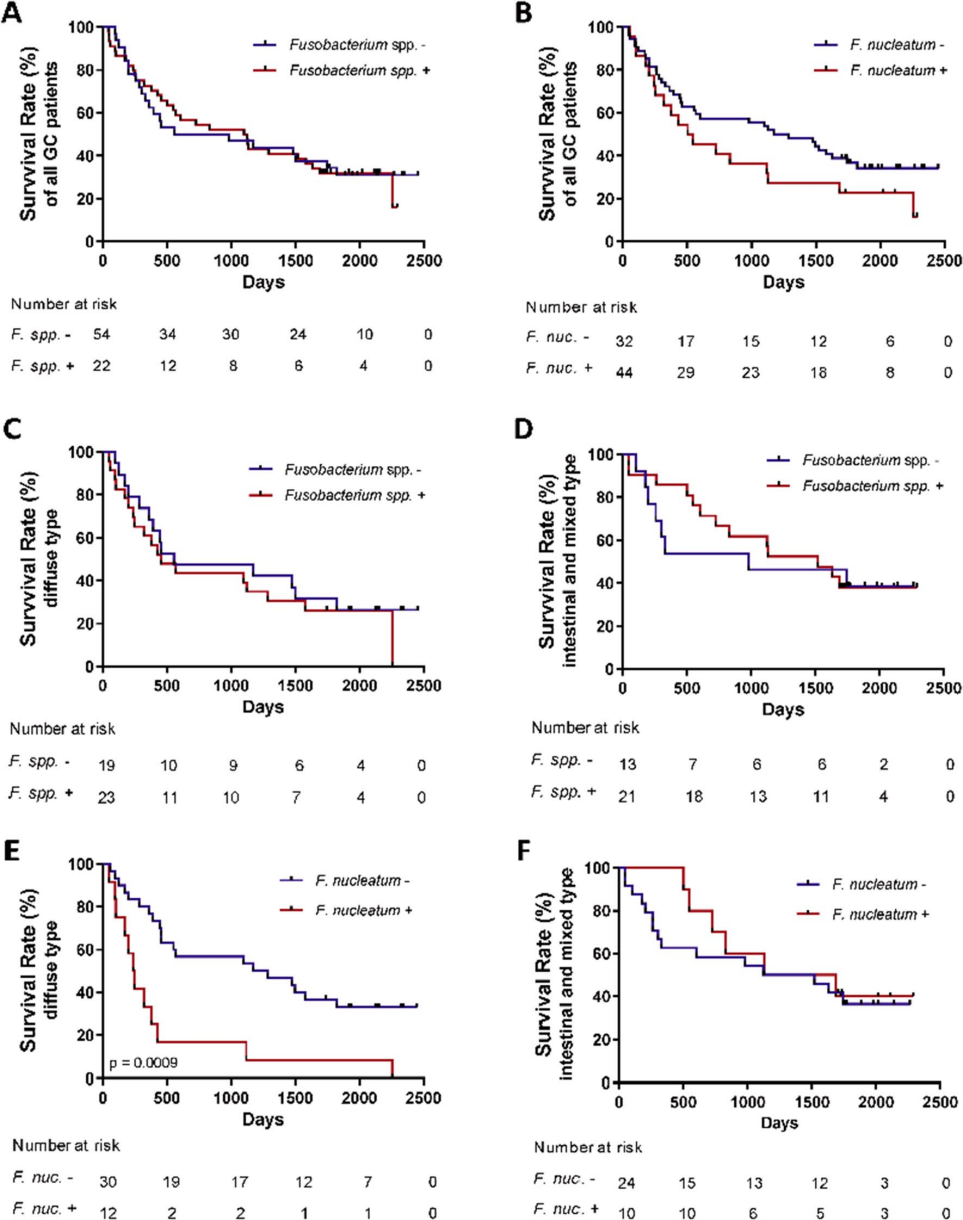
Overall survival rates of GC patients based on *Fusobacterium *spp*. and F. nucleatum* status. (**A**) Overall survival rates of GC patients with positive and negative *Fusobacterium* spp. status (p = 0.285). (**B**) Overall survival rates of GC patients with positive and negative *F. nucleatum* status (p = 0.129). (**C**) Overall survival rates of GC patients with Lauren’s diffuse type with positive and negative *Fusobacterium* spp. status (p = 0.536). (**D**) Overall survival rates of GC patients with Lauren’s intestinal and mixed types with positive and negative *Fusobacterium* spp. status (p = 0.798). (**E**) Overall survival rates of GC patients with Lauren’s diffuse type gastric cancer with positive and negative *F. nucleatum* status (p = 0.0009). (**F**) Overall survival rates of GC patients with Lauren’s intestinal and mixed types with positive and negative *F. nucleatum* status (p = 0.643). Log-rank (Mantel–Cox) test was used for survival data.

Survival data were obtained for GC subjects for a period of up to 2500 days. To avoid potential bias related to surgical complications, we excluded in total four patients from analysis due to the death within the first 30 days after receiving the diagnosis (1 with *F. nucleatum* positive and 3 with *F. nucleatum* negative T-GCs). Median survival of 76 patients was 981 days. Overall survival analysis revealed no difference between *Fusobacterium* spp. positive and negative gastric cancer patients (Fig. 5A, p = 0.997). In comparison (Fig. 5B), survival analysis based on the *F. nucleatum* positivity revealed a trend for a worse overall survival in the *F. nucleatum* positive group (524.5 days) in comparison to the *F. nucleatum* negative group (1287 days, p = 0.13). Survival analysis between different GC subgroups based on Lauren’s classification revealed no difference in regard to *Fusobacterium *spp. positivity both for diffuse and for intestinal or mixed-types of GC patients (Fig. 5C,D). Remarkably, survival of the patients with *F. nucleatum-p*ositive (n = 10) vs. –negative (n = 24) T-GC revealed no difference in the group with intestinal and mixed-type tumours (1406 vs 1323 days, p = 0.64, respectively) (Fig. 5E). However, patients with *F. nucleatum*-positive diffuse type of GC (n = 12) had significantly worse overall survival compared to *F. nucleatum*-negative (n = 30) GC (244.5 days vs. 1229.5, p = 0.009, respectively) (Fig. 5D). Comparison of clinicopathological characteristics of *F. nucleatum* positive and negative diffuse type of GC revealed only differences in age, but no other major differences, suggesting stage-independent effect of *F. nucleatum* positivity on the prognosis in diffuse type GC (Table 2).

**Table 2:** Comparison of the patients with *F. nucleatum* positive and negative Lauren’s diffuse subtype gastric cancer tumours.

	*F. nucleatum positive*		*F. nucleatum negative*		p
	n = 12	%	n = 30	%	
Age (years ± SD)	70.75	± 10.25	61.07	± 11.89	0.0189*
**Gender**					
Male	6	50.00	17	56.67	ns
Female	6	50.00	13	43.33	
**Tumor localization**					
Cardia	1	8.33	1	3.33	ns
Corpus	8	66.67	17	56.67	
Antrum	3	25.00	12	40.00	
**UICC**					
I	1	8.33	5	16.67	ns
II	4	33.33	6	20.00	
III	6	50.00	16	53.33	
IV	1	8.33	3	10.00	
**T**					
1 + 2	1	8.33	7	23.33	ns
3	5	41.67	15	50.00	
4	6	50.00	8	26.67	
**N**					
0	5	41.67	9	30.00	ns
1	4	33.33	5	16.67	
2	0	0.00	6	20.00	
3	3	25.00	10	33.33	
**M**					
0	10	83.33	27	90.00	ns
1	1	8.33	3	10.00	
Unknown	1	8.33	0	0.00	
**G**					
2	3	25.00	3	10.00	ns
3	9	75.00	27	90.00	

*F. nucleatum* positivity was defined by the cut-off of ≤ 38. *Unpaired t-test; ns—non-significant (p > 0.05).

## Discussion

Increasing evidence suggests that *F. nucleatum* may be involved in tumour development and associated with worse prognosis in CRC and other cancers. However, only limited data is available on the role of *F. nucleatum* in GC and gastric preneoplastic conditions. Using a well-characterized cohort of GC patients, we showed that *Fusobacterium *spp. and *F. nucleatum* may be frequently found not only in N- and T-CRC, but also in N- and T-GC although less frequently and at lower abundance. *F. nucleatum* was furthermore detected in normal mucosa and chronic gastritis. Interestingly, *F. nucleatum* was found in N-CRC and T-CRC in higher abundance despite the anatomical distance compared to N-GC and T-GC, respectively. Overall survival analysis revealed a significantly worse prognosis of patients with *F. nucleatum*-positive T-GC only in Lauren’s diffuse type GC, but not in intestinal type GC.

Amounting evidence has been collected to confirm the presence of *F. nucleatum* in CRC. Our data are in the frame of existing reports showing the *F. nucleatum* positivity in up to 60% of CRC specimens^8,11,26^. Only few preliminary reports have been dealing with this topic in GC and no data to prognostic relevance of *F. nucleatum* in GC has been studied yet. Yamamura et al. studied 20 samples from various GI cancers and detected *F. nucleatum* in 2 out of 20 cases^24^. Hsieh et al. demonstrated an enrichment of *F. nucleatum* in GC and have suggested *F. nucleatum* as potential diagnostic biomarker for GC^25^. In our cohort, we observed *F. nucleatum* positivity in GC patients in up to 28.75%. Surprisingly, the absolute abundance of *F. nucleatum* in T-GC was not different to N-GC, which is different to CRC studies.

We next performed the comparison of *F. nucleatum* absolute load between tumorous and non-tumorous colon and gastric mucosa. Both *Fusobacterium *spp. and *F. nucleatum* were at higher abundance in N-CRC compared to N-GC, as well as in T-CRC compared to T-GC. It is remarkable as the anatomical distance and proximity to an oral cavity would probably rather suggest higher abundance of *F. nucleatum* in the stomach as in the colon. Two reports have recently published results elaborating on the potential mechanism of *F. nucleatum* transfer to the tumours. Abed et al.^27^ have recently shown that host polysaccharide Gal-GalNAc, which is overexpressed in CRC, recognizes fusobacterial Fap2, which may trigger binding of *F. nucleatum* to the tumours. In another report, the authors confirmed an increased Gal-GalNAc levels in various tumours including GC^28^. Although the level of Gal-GalNAc was high in both CRC and GC tissues, the level in non-tumorous CRC samples was much lower as in non-tumorous GC which may explain the load differences. Overall, our results may support the hypothesis of potential hematogenous route of *F. nucleatum* spreading.

Some time ago, several initial studies have reported the capability of *F. nucleatum* to form biofilms. For instance, Zilm et al. reported that *F. nucleatum* may form biofilms and optimize its adhesion characteristics^29^. This property of *F. nucleatum* was dependent on the host environment in response to alkaline pH^30^. In CRC using the 3-dimensional tumour spheroid model, Kasper et al. observed development of biofilm-like structure in the tumour spheroid microenvironment by *F. nucleatum*^31^. The pathogenicity of *F. nucleatum* in the stomach may however be different as its low pH creates a unique microenvironment and microbial interplay. Low abundance of *F. nucleatum* in stomach in comparison to colon allows us to speculate on protective properties of acidic milieu preventing *F. nucleatum* dissemination. From another side, we observe no clear pattern for an increased abundance of *F. nucleatum* in AG/IM tissues where higher pH due to mucosa atrophy is expected. Considering the increasing interest in biofilm formation in the colon, further studies will be also necessary to address this point in the stomach microenvironment.

To understand the functional role of *F. nucleatum* in GC, we next analysed *F. nucleatum* in non-/preneoplastic gastric mucosa under consideration if *H. pylori* status and performed survival analysis. Aviles-Jimenez et al. have recently linked certain alterations in stomach microbiota composition to Correa’s cascade stages from CNAG to IM to intestinal type gastric cancer^32^. In our specific quantitative analysis, we did not observe any difference in *F. nucleatum* in preneoplastic conditions as well as no clear signal was found for *H. pylori* status. Since the sample size was sufficient only for pilot analysis, further studies will be needed to take a closer look at the *F. nucleatum* abundance in preneoplastic conditions with its variables and influencing factors specifically.

*F. nucleatum* has been repeatedly associated with worse prognosis in patients with oesophageal cancer^9^, pancreatic cancer^10^ and colorectal cancer^26,33^, but the data on GC are not available, yet. Although the overall survival analysis revealed only a non-significant trend toward a worse prognosis, we further performed subgroup analysis based on the Lauren’s classification, which is one of the most simple and valuable classifications of GC that partially mirrors the molecular GC classification and is frequently underappreciated in scientific work related to GC^34^. While no pattern was observed for intestinal type, we observed significantly worse overall survival in diffuse type GC patients with *F. nucleatum* positive tumours. It has been reported that *F. nucleatum* may promote carcinogenesis in CRC via FadA adhesin, which binds to E-cadherin, activated β-catenin signalling and accordingly various inflammatory and oncogenic properties of the cells^35^. Since diffuse type of GC is strongly associated with E-cadherin deregulation one may speculate for potential molecular mimicry of *F. nucleatum* to diffuse type of GC and probably specific prognostic relevance.

In one of the pivotal reports, *F. nucleatum* was associated with CIMP positivity, hMLH1 methylation, MSI and CHD7/8 positivity^11^. We analysed correlation between *F. nucleatum* and LINE-1 as a global methylation marker and miR-137 methylation^36^. *F. nucleatum* positive GC tumours showed a trend to lower LINE-1 methylation with overall positive correlation, while no association was found for miR-137. Although this may suggest that indeed, *F. nucleatum* positivity could be associated with certain epigenetic alterations such as global DNA hypomethylation, from another side, the lower LINE-1 DNA methylation could also be related to the aging as *F. nucleatum* positivity correlated strongly also to older age.

Despite intriguing results, we would like to underline that this is one of the first analyses and multiple remaining questions need to be addressed in future work. First, the study aimed to evaluate specifically the translational role of *F. nucleatum* in GC, therefore the data acquired may allow only a partial view on the microbial changes. Microbiome-sequencing may provide in-depth view on microbial alterations in GC. Second, our work provides only some preliminary molecular analysis on correlation with LINE-1 methylation. Additional in vitro and in vivo studies should provide mechanistic insights and explanation. Third, in particular from the clinical point of view, the data to *F. nucleatum* may have substantial clinical consequences. It has been recently reported that antibiotic treatment of tumours harbouring *F. nucleatum* led to reduced tumour growth in mice^12^. Therefore, use of antibiotics (for example metronidazole) could be a possible therapeutic consequence in patients with diffuse type GC with *F. nucleatum* positivity. Furthermore, the impact of *Fusobacterium* on the treatment response especially in the era of immunotherapy may be quite intriguing. Recently, it has been reported that prudent diets rich in whole grains and dietary fibres were associated with lower risk of *F. nucleatum* positive CRC while diets that may promote intestinal inflammation were associated with increased risk of *F. nucleatum* positive tumours^37,38^. Diet has been shown to provide a great source of various microRNAs including xenomiRNAs^39^, therefore, taking into account an association between diet and *F. nucleatum* positivity one may speculate on the role of exogenous microRNA or even various drugs. Further studies will be needed to address the impact of proton-pump-inhibitors and antibiotics on positivity and variation of *F. nucleatum* in stomach and CRC.

In summary, the results of our work strongly support the potential involvement of *F. nucleatum* in gastric carcinogenesis. *F. nucleatum* is frequently found in normal, preneoplastic and neoplastic mucosa although substantially lower than in colon. Even though there were no specific clinicopathological differences related to *F. nucleatum* positive gastric cancer patients, *F. nucleatum* positivity was associated with significantly worse overall survival in diffuse Lauren’s type GC patients. Further studies are needed to evaluate possible therapeutic implications and molecular alterations responsible for this phenotype.

## Materials and methods

### Study design

Prospectively collected samples were evaluated in this study. Tissue samples were collected in the Departments of Gastroenterology and Surgery at the Hospital of Lithuanian University of Health Sciences (Kaunas, Lithuania) and in the Department of Gastroenterology, Hepatology and Infectious Diseases at the Otto-von-Guericke University Magdeburg (Germany) in the context of the ERA-Net PathoGenoMics project. The study was performed according to the principles of the Declaration of Helsinki. Kaunas Regional Bioethics Committee (No. BE-2-10) and Institutional Review Board of Otto-von-Guericke University Magdeburg (No. 80/2011) approved both studies. All patients participating in the study provided written informed consent.

### Survival analysis

The Lithuanian Cancer Registry and the Hospital of Lithuanian University of Health Sciences collected survival data of the gastric cancer patients for up to 2500 days. The time of survival was measured as the time interval between the date of GC diagnosis and the date of death.

### Samples collection

The collection and characterization of biological material was partly described in our previous studies^36,40^. Briefly, specimens from GC and CRC were prospectively collected during surgical interventions. Samples from controls (N) and patients with various stages of chronic gastritis were obtained during endoscopy. The samples were immediately snap-frozen in liquid nitrogen and placed in − 80 °C freezer. The updated Sydney classification was applied for histological characterization of gastritis^41^. The Lauren’s classification was used for histological assessment of GC tumours. *H. pylori* status was analysed either by *H. pylori* ELISA IgG test (Virion\Serion GmbH, Germany) for GC patients or using multistep approach via serology, microbiology and histology as previously reported^42,43^. We obtained 81-paired samples from patients with GC including tumour tissues (T-GC) and their corresponding adjacent non-tumorous gastric mucosa (N-GC). Histopathological assessment of GC tissues was performed by an experienced pathologist at the tertiary centre form Lithuania. For preliminary analysis we included samples from 18 patients with histologically confirmed normal gastric mucosa (N), 17 patients with CNAG and 9 patients with AG/IM. In addition, we included samples from 27 patients with colorectal cancer (T-CRC) and their corresponding adjacent non-tumorous colon mucosa (N-CRC). An overview for sample collection and methods are presented in Supplementary Table S1 and the clinical and demographic data in Table 1.

### DNA isolation and quantitative real-time PCR

DNA was extracted from frozen tissue samples, pretreated with QIAzol Lysis reagent (Qiagen, Valencia, CA) and chloroform based on manufacturer’s recommendations as described previously^36,40^. Probe-based quantitative real-time PCR was performed using Bio-Rad CFX96 real-time PCR cycler (BioRad, CA). Following probe-based primer were used: *Fusobacterium *spp.^44^; *F. nucleatum*^9^; prostaglandin transporter (PGT), also known as solute carrier organic anion transporter family, member 2A1 (SLCO2A1), as endogenous control for normalization as previously described^8^. Primer and probe sequences are provided in Supplementary Table S2. Ct-values for *Fusobacterium *spp. and *F. nucleatum* were set to 40 if PCR analyses revealed a negative result. Normalization was performed using 2^deltaCt-method. The values of the samples with undetectable *Fusobacterium *spp. and *F. nucleatum* were set to the lowest measurable normalized values.

### Methylation analysis

Purified genomic DNA from tissue samples was used for global long interspersed nucleotide element-1 (LINE-1) and miR-137 promoter methylation analyses. The procedure was in detail described in our previous reports^36,40^. Briefly, we applied Cells-to-CpG Bisulfite Conversion Kit (Life Technologies, Carlsbad, CA) for bisulphite modification, thereafter the standard PCR with biotin-labelled primers and eventually the pyrosequencing on PyroMark Q96 ID (Qiagen) using PyroMark Gold Q96 reagents (Qiagen). The mean methylation level of analysed CpG motifs was used for quantitative methylation analysis.

### Statistical analysis

Statistical evaluation was conducted with GraphPad Prism 7.0 (San Diego, CA), statistical software. We applied χ^2^-test for qualitative analysis and for quantitative analysis we used either Wilcoxon test for paired samples or Mann–Whitney U test for unpaired samples. For comparison of more than two groups we used the Kruskal–Wallis test. Spearman’s test was applied for correlation analysis. Survival analyses were performed with the Mantel-Cox test. Two-sided *p*-values of < 0.05 were considered as statistically significant.

### Ethical standards

The study was performed according to the principle of the Declaration of Helsinki. The study was approved by the Kaunas Regional Bioethics Committee No. BE2-10 and Institutional Review Board of Otto-von-Guericke University Magdeburg No. 80/2011. All patients provided written informed consent.

## Supplementary information

Supplementary file 1.

## Data Availability

The data that support the findings of this study are available from the corresponding author upon reasonable request.
