# Supplementary material for: Fusobacterium nucleatum is associated with worse prognosis in Lauren’s diffuse type gastric cancer patients
Source: Sci Rep. 2020 Oct 1;10:16240. doi: 10.1038/s41598-020-73448-8 (PMC7530997; doi:10.1038/s41598-020-73448-8)
Supplement: Supplementary file 1 [file 41598_2020_73448_MOESM1_ESM.pdf]

Original Article

Fusobacterium nucleatum is associated with worse prognosis in Lauren´s diffuse type gastric cancer patients

Ellen Teresa Boehm[1], Cosima Thon[1], Juozas Kupcinskas[2,3], Ruta Steponaitiene[2], Jurgita Skieceviciene[2], Ali Canbay[1], Peter Malfertheiner[1] and Alexander Link[1]

[1]Department of Gastroenterology, Hepatology and Infectious Diseases, Otto-von-Guericke University Magdeburg, Magdeburg, Germany

[2]Institute for Digestive Research, Lithuanian University of Health Sciences, Kaunas, Lithuania

[3]Department of Gastroenterology, Lithuanian University of Health Sciences, Kaunas, Lithuania

Corresponding author
Alexander Link, M.D., Ph.D.
Department of Gastroenterology, Hepatology and Infectious Diseases
Otto-von-Guericke University
Leipziger Str. 44
39120 Magdeburg, Germany
Phone: +49-391-6713745
Fax: +49-391-6713105
Email: alinkmail@gmail.com or alexander.link@med.ovgu.de

Supplementary Table S1: Overview of samples and methods

**N of patients**	**Group description**	**Methods**			
		Fusobacterium spp.	*F. nucleatum*	Overall survival analysis	LINE-1 and miR-137
**81**	Total number of patients with GC included in the study (N-GC and T-GC)	X	X	X	X
**80***	Total number of patients with available *F. nucleatum* status	X	X	X	X
**27**	Total number of patients with CRC included in the study (N-CRC and T-CRC)	X	X		
**18**	Patients with normal gastric mucosa (N)	X	X		
**17**	Patients with chronic non-atrophic gastritis (CNAG)	X	X		
**9**	Patients with atrophic gastritis with and without intestinal metaplasia (AG/IM)	X	X		

* one T-GC sample was lost during analysis

Supplementary Table S2: Primer sequences

Name	Primer sequence	Annealing temperature
**Prostaglandin transporter (PGT)** [11]	F 5‘-ATC CCC AAA GCA CCT GGT TT-3‘ (20 bp)<br>R 5‘-AGA GGC CAA GAT AGT CCT GGT AA-3‘ (23 bp)<br>Probe 5‘-CCA TTC ATG TCC TCA TCT C-3‘ (19 bp)	59°C
Fusobacterium spp. [41]	F: 5‘-GGA TTT ATT GGG CGT AAA GC-3‘ 20 bp)<br>R: 5‘-GGC ATT CCT ACA AAT ATC TAC GAA-3‘ (24 bp)<br>Probe: 5‘-CTC TAC ACT TGT AGT TCC G-3‘ (19 bp)	59°C
F. nucleatum [9]	F 5’-TGG TGT CAT TCT TCC AAA AAT ATC A-3’ (25 bp)<br>R 5’-AGA TCA AGA AGG ACA AGT TGC TGA A-3’ (25 bp)<br>Probe 5’-ACT TTA ACT CTA CCA TGT TCA-3’ (21 bp)	54°C

Supplementary Figure S1

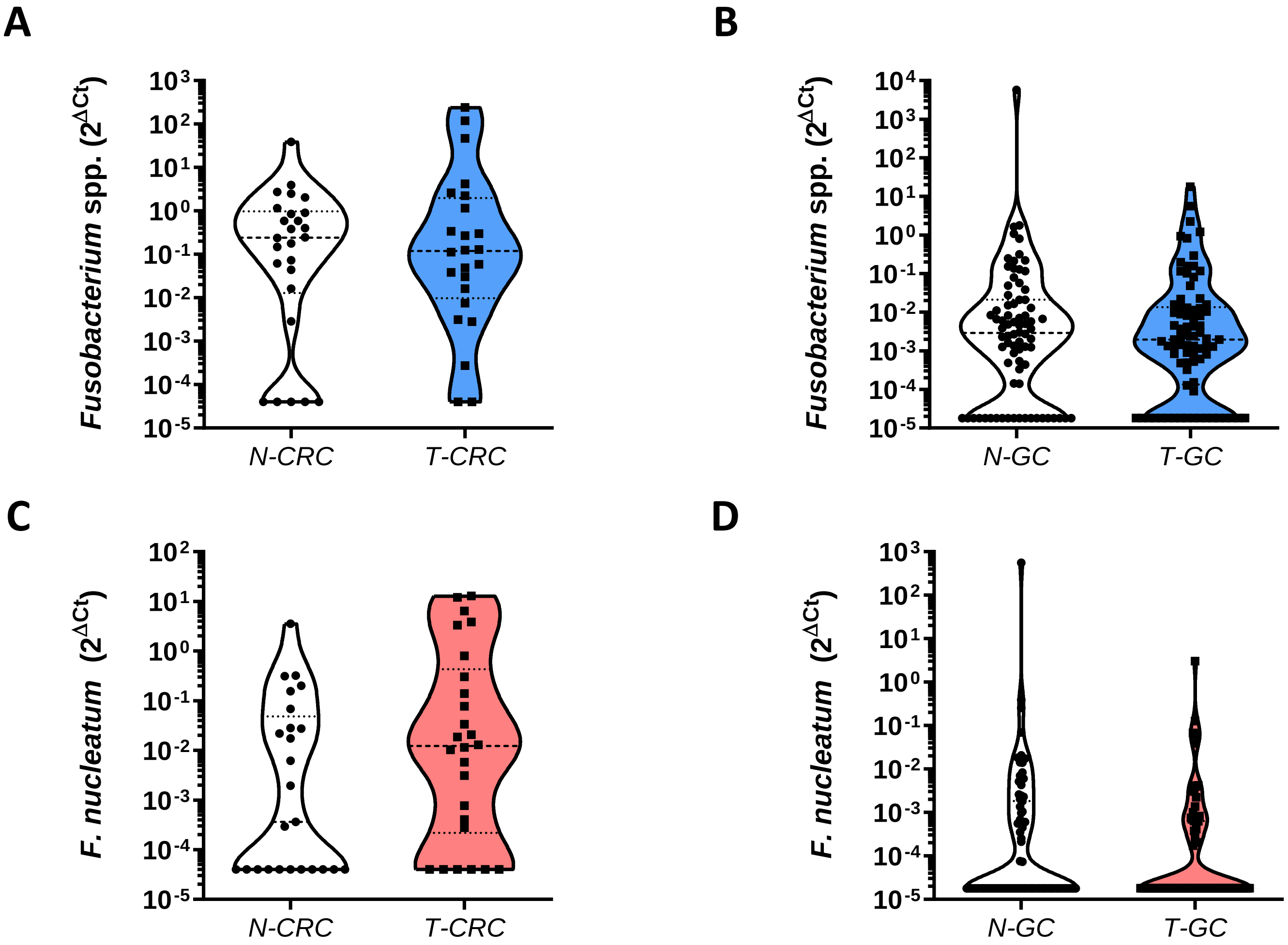

Supplementary Figure S1. Relative abundance of *Fusobacterium spp.* and *F. nucleatum* in colorectal and gastric cancer patients. (A) Relative abundance of *Fusobacterium spp.* in N-CRC (n=26) and T-CRC (n=24). (B) Relative abundance of *Fusobacterium spp.* in N-GC (n=78) and T-GC (n=80). (C) Relative abundance of *F. nucleatum* in N-CRC (n=25) and T-CRC (n=26). (D) Relative abundance of *F. nucleatum* in N-GC (n=79) and T-GC (n=80). Relative abundance is presented as $2^{\Delta CT}$ values normalized to PGT. Undetectable values were set to the lowest measurable normalized value. Mann-Whitney-test was used for statistical analysis of two groups and Kruskal-Wallis test for more than two groups.

Supplementary Figure S2

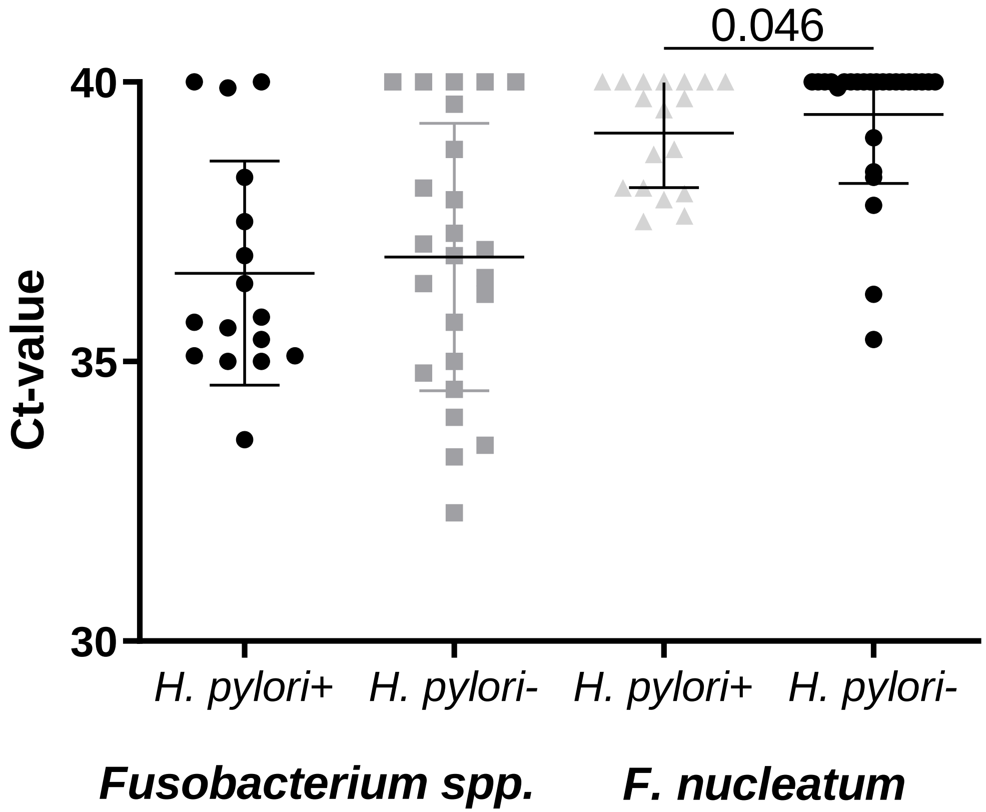

Supplementary Figure S2. Analysis of *Fusobacterium spp.* and *F. nucleatum* abundance in relation to *H. pylori* status in controls and chronic gastritis patients. *H. pylori* positive group includes the patients with CNAG, AG/IM while *H. pylori* negative groups includes also the control group. Mann-Whitney-test was used for statistical analysis.
